# Power motivation arousal promotes prosocial behavior in the dictator game depending on social presence

**DOI:** 10.1371/journal.pone.0277294

**Published:** 2022-11-04

**Authors:** Jianfeng Wang, Shuangyi Qu, Ruiyu Li, Yunqiao Fu

**Affiliations:** 1 School of Psychology, Chengdu Medical College, Chengdu, China; 2 Sichuan Research Center for Applied Psychology, Chengdu Medical College, Chengdu, China; Universidad de Murcia, SPAIN

## Abstract

Despite the popular notion that power motivations are associated with aggression and antisocial behavior, this study tested the hypothesis that activating power motivations can promote prosocial behavior. Because previous research has shown that public prosocial behavior is associate with reputation and status, this study examined how making prosocial decisions publicly or privately moderates the relationship between power motivations and prosocial behavior. One hundred and forty participants were randomly assigned to watch 20 min of either *The Experiment* (power motivation arousal) or a documentary called *Beautiful China* (control condition). A modified version of the dictator game was used to measure prosocial behavior. Participants were instructed to allocate an amount of money between themselves and a stranger girl in need, in the presence of the experimenter (the experimenter registers donation amount) or in the absence of the experimenter (the donation was put in a closed envelope). The results showed that individuals in the power motivation arousal group increased their help when their reputation was under scrutiny due to the experimenter’s presence. In the private condition (experimenter is absent), power motivation is not related to prosocial behavior. The contrasting behavioral reactions resulting from the presence or absence of the experimenter are discussed in terms of reputation gain and competitive altruism.

## Introduction

Power is a fundamental and essential element of human society. Russell claimed that “love of power is the chief motive producing the changes that social science has to study” [1]. This study contributes to our understanding of this key motivation in human behavior. Environmental factors should be considered when analyzing motivations as the driving force behind human behaviors. This is necessary because specific external conditions can interfere with and alter motivated behavior [2]. It is necessary to consider motivations and the situation in which the person acts to understand why a person does what they decide to do [3]. This study focused on the interaction between a specified motivational factor (power motivation) and one specified situational factor (social presence).

In the following section, a brief discussion is provided on the two aspects of power motivation. These discussions highlight how power-motivated individuals are particularly concerned about their reputations. Researchers have taken several different approaches in the study of power motivation. One method is to place participants in environments with motivational incentives and measure changes in physiology or behavior as a function of the motivational situation, as in this study, participants were exposed to movie clips filled with cues of power and dominance. Moreover, researchers can also attempt to measure motivation through individual differences in one’s personality. Therefore, in this study, we use the term "power motivation" to denote an experimentally induced motivational state and the term "power motive" to denote a stable trait. Next, reputation has been discussed as a critical factor in determining an individual’s tendency to behave prosocially in the presence of others. Finally, one study is reported testing our main hypotheses.

### The two faces of the power motivation

According to McClelland [4], the power motive (also called the need for power) is one of the main categories of human motivation. Power motive is the tendency to be pleased by having influence over other individuals and displeasure at potential external influence on oneself [5, 6]. Power-motivated individuals are especially concerned with their social status, reputation, and prestige [4]. They emphasize their importance in social activities by drawing attention to themselves and attempting to impress others [7]. The power motive was predominantly recognized as the underlying cause for obstinate, offensive, and reprehensible behaviors, destructive to society [8]. For example, the power motive was a significant predictor of risk-taking in gambling, autocratic business decisions, and aggressiveness [7, 9–11]. In a money distribution task, participants with high power motive were less likely to share their money with other virtual players and allocated more money to themselves [12]. Yost and Zurbriggen [13] reported that high-power men engaged in unrestricted sociosexual activities. McClelland [14] found that high-power women preferred open relationships that allowed them to have sexual contact with people other than their partners. Based on this, it is unsurprising that common perceptions of power motive were quite negative.

Despite the academic and the public perception of power motive as an undesirable social factor, it also contains innate benevolent and prosocial characteristics. Researchers such as McClelland [4] and Winter [6] have already indicated the duality of power, describing how people might express power motivation in either an antisocial or prosocial direction. The core concept of the need for power emphasizes the influence, not domination, of others [5]. Power stereotypes are generally aggressive and authoritarian. However, among many mammalian species, particularly primates, exhibiting aggressive and authoritarian behavior is rarely a sustainable strategy for attaining and maintaining dominance [15]. Such behavior does not typically characterize individuals with high levels of power motive [5]. Existing research has shown that groups punish members who seek privileged status through violence and aggression [16].

Power-motivated individuals often seek to influence others using a more perspicacious approach. Instead of self-serving or egotistical actions, it is socially preferable behavioral strategies that allow power-motivated individuals to achieve their goals. Egocentric behavioral strategies can impress individuals with antisocial characteristics such as imprudence and arbitrariness. Subsequently, egocentric behavioral strategies make achieving long-term stable societal influence extremely difficult. Such strategies are in direct opposition to the goals of power-motivated individuals. Ditlmann, Purdie-Vaughns, Dovidio, and Naft [17] found that high-power motive, oppressed minority members adopted socially engaging, warm style communication strategies when talking to majority group members. These strategies meant that power-motivated minority members had a greater impact on their audiences. This suggests that power-motivated people cannot simply concentrate on enforcing their dominance. For social success, they must consider flexible and adaptive strategies in response to specific circumstances to stabilize maximal positive influence [18, 19].

### Social presence

Research has shown that social presence has a significant influence on prosocial behavior. For example, participants allocated much more money when the experimenter observed their decision than when they were anonymous in the dictator or ultimatum games [20]. When the participants realized their actions were being displayed publicly, their donations in the public goods game increased [21]. It was also shown that more participants would choose to help once they knew that benevolent actions were recognized by others [22]. More than the actual presence of others, even very subtle cues of being watched (i.e., stylized watching eyes) may influence the decision to help [23–25].

Reputation is generally recognized as the key factor in encouraging prosocial behavior in terms of social presence. A person’s prosocial behavior is often motivated by the expectation of an increased positive reputation, which is beneficial to social interactions [26, 27]. An individual’s reputation as a cooperative and helpful group member is especially advantageous in the long term. People with a reputation are often considered highly reliable and charming and are more likely to be considered when selecting friends, team members, and romantic partners [28, 29]. Importantly, being prosocial without being obsequious is associated with status in a group [21].

It should be assumed that people who are aroused with power motivations would intentionally act prosocially due to the status-elevating benefits of cooperation. For those who aspire to influence, expressing their power motivation in the form of prosocial behavior is advisable. Few studies have investigated the moderating effects of contextual factors on the relationship between the power concept and prosocial behavior. Conlon and Rose [30] found that power priming decreased people’s environmental attitudes and willingness to sacrifice for the environment, but only when their responses were private. Kraus and Callaghan [31] found that the relationship between social class and prosocial behavior varies in public and private contexts. Low class participants were more prosocial in private than in public, whereas upper class participants exhibited the opposite pattern. Furthermore, Griskevicius, Tybur, and Van den Bergh [32] found that status motives led people to abandon luxuries and desire prosocial environmental products only when such choices can be observed. In a recent study involving power motive, Wang and Dai [33] found that individuals with higher power motive exhibited stronger prosocial tendencies when influenced by social presence. One limitation of this study is the correlative nature of the relationship between power motivation and prosocial behavior. Further research is required to investigate whether there is a causal relationship between power motivation and prosocial behavior. Researchers should experimentally arouse power motivation to examine whether this influences prosocial behavior. Another limitation is the use of the ultimatum game to measure prosocial behavior. In the ultimatum game, the proposer and responder allocate funds. The proposer decides on an allocation scheme, and the responder chooses to accept or reject it. If the responder chooses to accept, the funds are distributed as proposed; if they reject, both sides receive nothing [34]. Previous studies have shown that power-motivated individuals are sensitive to power threats [35–38]. The proposer’s allocation decision in the Ultimatum Game may be based on fairness. However, it may also be that people with high power motive are more concerned that the other party may reject an unfair allocation scheme.

### Overview of the current research

The present study used a modified dictator game to overcome these limitations [39]. In the present study, the dictator is asked to allocate a fund between herself/himself and a stranger girl in need to measure prosocial behavior. In the dictator game, the proposer can distribute the amount in any way he/she chooses, and the responder must accept the allocation. Therefore, in this paradigm, there are no power threats. Moreover, we presented participants with movie excerpts designed to arouse their power motivation. This method has been successfully utilized in previous studies [40]. Specifically, excerpts with strong power and dominant content were chosen to arouse the power motivation in participants. The control group watched a documentary with motivationally neutral content. Social presence was manipulated according to whether the experimenter observed the participants’ charitable donations. Based on the above theoretical considerations, we hypothesized that participants in the power motivation arousal group might be less likely to help when the experimenter was absent but would be more willing to help when the experimenter was present.

## Method

### Participants and design

Our experiment sample size was set according to *a prior power analysis* using G*Power 3.1.9 [41]. Based on medium effect size (f^2^ = 0.15), power (1-β error probability) of 0.95, and α error probability of 0.05, the power analysis yielded an estimated sample size of 119. Data were collected from 140 undergraduate students (77 women, 63 men; mean age 19.59). The study was approved by the local review board for research involving human participants. All participants provided written informed consent before participating in the experiment.

A 2×2 (arousal condition × social presence) between-subjects design was adopted with 35 participants in each experimental condition (see Table 1). The arousal condition included the power motivation arousal group and the neutral control group. Social presence included the presence or absence of the experimenter. The dependent variable was the money allocated by the dictator to the stranger girl in need.

**Table 1:** Experimental grouping and the number of subjects in each cell.

Social presence	Arousal condition		Total
	Power motivation	Control	
The presence of the experimenter	35	35	70
The absence of the experimenter	35	35	70
Total	70	70	140

### Procedure

A cover story was used to minimize participants’ suspicions. Upon arriving at the lab, participants were told they would complete several different studies, the first of which was to rate a movie excerpt for likability. Consistent with this cover story, participants watched an excerpt of the movie and rated it. Subsequently, the participants were told that they were going to participate in a second study. The instructions were as follows.

“Please complete the questionnaire shown in the table. After that, please open the envelope in the upper right corner of the table. It contains the fee we will pay you. We want to inform you that we are starting a fundraiser for a girl at our college. She comes from a poor family and was recently involved in a car accident. There is RMB 10 in the envelope. You can donate as much as you like and take the rest with you as thanks for your participation. ‘After making your donation, seal the envelope and drop it into the donation box. Your donation amount will be kept confidential [experimenter’s absence].’ OR ‘After making your donation, please give the envelope to the experimenter, and he will register your donation amount [experimenter’s presence].’”

Participants then completed two manipulation checks: ‘someone has already looked at my allocation decision’ or ‘my allocation decision has been kept private’ (1 = strongly disagree, 5 = strongly agree). Finally, they were thanked and debriefed.

### Measures

#### Arousal condition

Participants in the power motivation arousal group watched a clip of about 20 minutes from the movie, *The Experiment*. The movie was adapted from the Stanford prison experiment. The excerpt shows correctional officers exerting authority by punishing prisoners and experiencing the thrill of power through respect and fear. The excerpt is loaded with themes of power, domination, and influence. Participants in the neutral control group watched a 20-minute documentary called *Beautiful China* which depicted China’s wildlife and natural landscapes and was devoid of power cues.

#### Manipulation check

The Multi-Motive-Grid (MMG) [42] was used to evaluate whether the movie induced a power-motivational state and to measure the power motivation level after the movie. MMG is a widely applicable measurement tool in social motive studies [2, 43]. It consists of 14 pictures depicting daily situations, each accompanied by several statements describing thoughts, feelings, and actions. In response to each statement, participants are asked whether they agree with the corresponding description (“Yes” or “No”). The MMG evaluates the hope and fear components of motives. To be comparable with other precedent motive research [33] that focuses on the approach component of motives, we evaluated the hope component of the power motive (hope for power, for example, trying to influence others). Motive scores ranged from 0 to 12. Cronbach’s alpha for the present study was 0.78.

#### Measurement of emotion

The Positive and Negative Affect Scale [44] measured participants’ emotions and excluded emotional influences on prosocial decision-making. The items were rated on a scale from 1 (very slightly or not at all) to 5 (very much). This study’s internal consistency reliability of positive and negative emotions was 0.87 and 0.83, respectively.

## Results

### Manipulation checks

The scores of power motivation in the power motivation arousal group (*M* = 8.61, *SD* = 2.15) were significantly higher than those in the neutral control group (*M* = 4.64, *SD* = 2.94), *t* (138) = 9.12, *p* < 0.001, Cohen’s d = 1.54, indicating that manipulation of power motivation is effective. Compared to participants in the private condition (*M* = 1.36, *SD* = 0.48), participants in the presence of the experimenter condition (*M* = 4.44, *SD* = 0.61) were more likely to agree that their donation decision had been looked at by the experimenter, *t* (138) = 33.35, *p* < 0.001, Cohen’s d = 5.64. Moreover, compared to participants in the presence of the experimenter condition (*M* = 1.21, *SD* = 0.41), participants in the private condition (*M* = 4.41, *SD* = 0.69) were more likely believe that their donation decision had been kept private, *t* (138) = 33.24, *p* < 0.001, Cohen’s d = 5.62.

### Preliminary analysis

Overall, the average donation was *M* = 3.84 (*SD* = 3.13). 73.6% (N = 103) of the participants wanted to donate some money to the stranger girl. There were no significant differences in the average amount of donated (*M*_men_ = 3.46, *SD* = 2.91; *M*_women_ = 4.16, *SD* = 3.28; *t* (138) = –1.31, *p* > 0.19) and proportion of willing to donate (*M*_men_ = 71.43%, *M*_women_ = 75.32%, χ^2^ = 0.27, *p* > 0.60) between different sexes. Moreover, the gender did not show any interaction with the independent variables and thus will not be discussed further. A 2 (arousal condition) ×2 (social presence) ANOVA for positive and negative emotions showed no significant main effects or interaction, indicating that there is no difference in emotional states under different conditions.

### Amount donated

**Fig 1:**
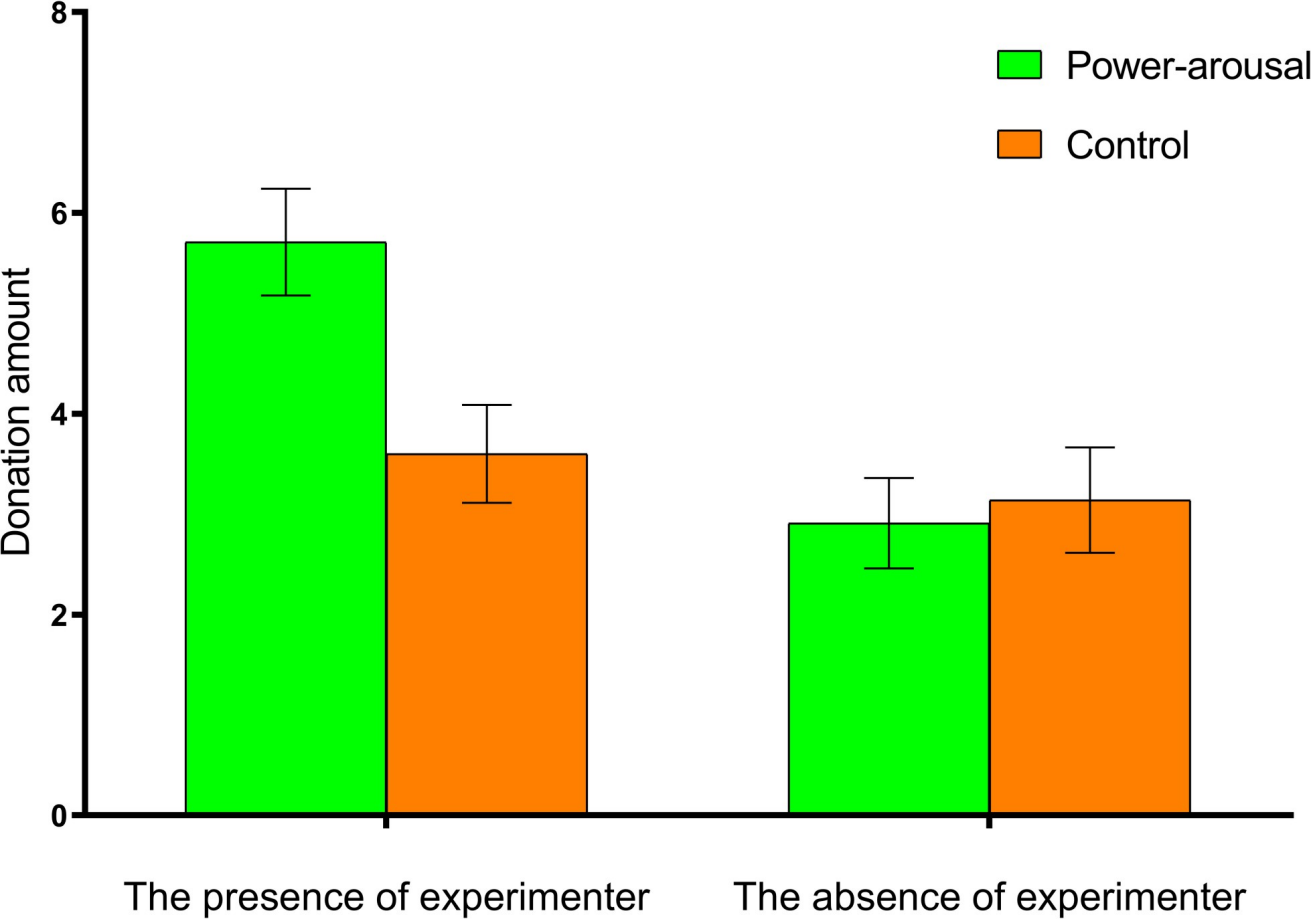
Charitable donation as a function of power motivation and social presence.

The 2 (arousal condition) × 2 (social presence) ANOVA yielded no significant effects of arousal condition, *F*(1, 136) = 3.57, *p* = 0.06, but did yield significant main effect of social presence, *F*(1, 136) = 10.64, *p* = 0.001, η = 0.07, and arousal condition × social presence interaction, *F*(1, 136) = 5.51, *p* = 0.02, η = 0.04. Specifically, in the presence of the experimenter condition, participants in the arousal group (*M* = 5.71, *SD* = 3.15) donated more than those in the control group (*M* = 3.60, *SD* = 2.88), *t* (68) = 2.93, *p* = 0.005, Cohen’s d = 0.70 (see Fig 1). In the absence of the experimenter condition, the difference between arousal group (*M* = 2.91, *SD* = 2.66) and control group (*M* = 3.14, *SD* = 3.01) was not significant, *t* (68) = 0.33, *p* = 0.74, Cohen’s d = 0.08.

### Willingness to donate

**Fig 2:**
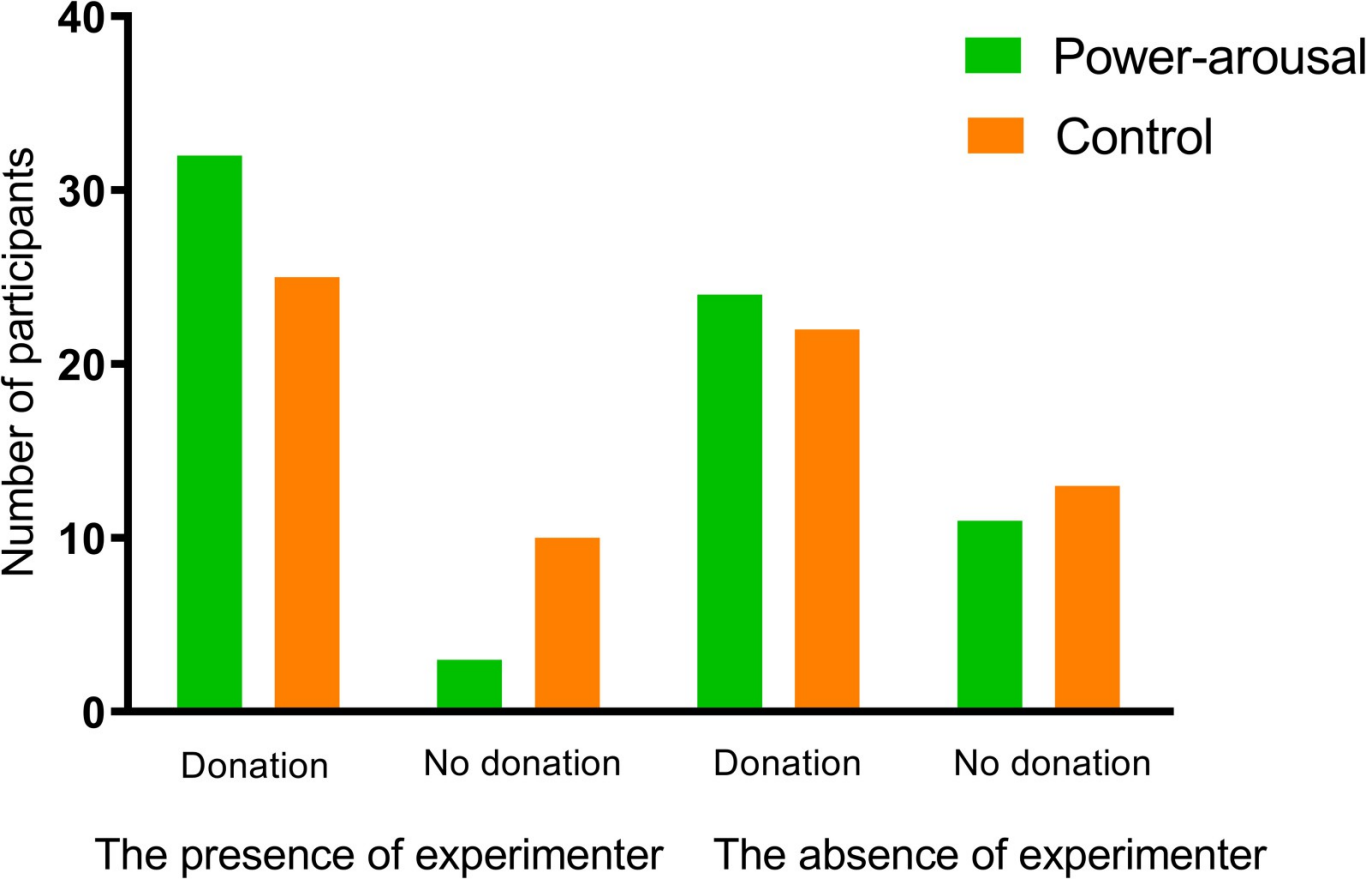
Number of participants from the power motivation arousal and control group who offered/did not offer donation in conditions that the experimenter present or not present.

As shown in Fig 2, due to the presence of the experimenter, participants in the arousal group were more likely to offer donation than those in the control group (32/35 vs. 25/35, χ^2^ = 4.63, *p* = 0.03), whereas in the absence of the experimenter they offer donation with a close to equal probability (χ^2^ = 0.25, *p* = 0.62).

## Discussion

The present study investigated the degree to which the tendency to behave in a selfish or prosocial manner in a dictator game is a function of power motivation and social presence. Although power motivations have been associated with selfishness, human motivation research implies that a strong power motivation is also likely to encourage prosocial and altruistic behavior [4, 6]. This theoretical hypothesis requires more empirical verification.

To better understand the dual nature of power motivations, we turned to the theory of competitive altruism, which suggests that prosocial behavior is linked to status or prestige [21, 45]. Competitive altruism theory posits that people competing to behave more generously than others in the presence of others. Previous work showed that the expression of a benevolent attitude could be an effective means of status elevation in their group. The key factor is a demonstration of concern for public benefits and a willingness to act. For example, individuals who contribute more to public funds are seen as more committed to the group and thus more likely to achieve higher status and stronger influence [21]. Accordingly, status seekers may adopt competitive altruism by attempting to overcome others with generosity [21, 45]. It does not appear that status-driven individuals achieve high status through bullying and intimidation. Previous research has shown that members who gain status through force and aggression are often punished by the group [16]. Activating power motivation may lead individuals to exhibit generosity when they expect reputational rewards related to altruism. The underlying reason is that when the individual’s decisions receive publicity, the potential benefits of being socially advocated for in future activities exceed the cost of present generosity.

Supporting predictions, our results suggested that activating power motivations led participants to be more sensitive to social presence cues. Individuals in the power arousal group donated more in the presence of the experimenter compared to when the experimenter was not present. Still, they tended to act according to egocentric principles when others could not observe them [46]. This finding aligns with previous results that found high power motive individuals are more likely to act fairly or cooperate in public situations [33]. These results suggest that activating power motivations enables individuals to both directly and forcefully influence others as well as strategically adapt to specific circumstances [17–19].

### Theoretical and practical implications

The results of this study have important implications for understanding how power motivation influences behavior. Previous research has found that power motives are associated with prosocial and antisocial decision-making [9, 12, 13, 33]. Our findings show that situational factors (i.e., social presence) moderate the effect of power motivation. It is well possible that the inconsistency could be a consequence of variations in the situational cues involved in the respective studies. Furthermore, the findings of this study may have implications for the behavioral approach theory of power, which argues that powerful individuals are agentic and tend to approach rewards and desirable goals [47]. For those whose power motivations are activated, giving up material benefits may not be a desirable goal. However, when they are observed in public, they may forego material benefits in exchange for a prosocial image and potentially higher social status. This suggests that, in pursuit of the outcome that is most personally beneficial, they exhibit an adaptive strategy shift when confronted with goal conflict situations.

This study may have practical implications for promoting prosocial behavior. Although power motive has traditionally had a bad reputation, our research suggests that arousing power motivation may be an effective strategy for promoting prosocial behavior. The key to motivating the prosocial side of the power motivation, however, is that prosocial behavior is openly visible to others. Therefore, it is best for social institutions or organizations to give their donors or volunteers some visible signs or labels that can convey reputation.

### Alternative explanation

A potential alternative explanation for our results is the experimenter demand effect [48]. If participants realized the true objectives of the experiment, then the results may be explained by an experimenter demand effect. This effect causes participants to behave in a way they believe the experimenter wants them to behave. In this regard, we first emphasize that the experimenter’s presence is a treatment variable. We deliberately designed it to convince participants that the experimenter was observing their behavior, thereby inducing reputational concerns. Moreover, participants completed a series of questionnaires after watching the movie clip to reduce the connection with the real experimental purpose. This effectively prevented the participants from guessing the experiment’s true objective. In the debriefing phase, we asked participants whether they thought about what we wanted them to do while they were participating in the task. The vast majority of the participants responded that they did not think about what the experimenter wanted them to do. A small number of participants made wrong guesses about what the experimenter wanted (like "to investigate our imaginations or emotions"), and none made guesses related to power motivation or prosocial behavior. Although the dictator game can examine the influence of situational factors on decision-making, it must be acknowledged that it is an artificial situation that may differ from the real-life environment. Therefore, future research should examine altruistic behavior in real-life situations.

### Limitations and future perspectives

This study had several limitations. First, although the power motivation priming manipulation performed in this study using movie excerpts [40] effectively aroused individuals’ power motivation, we could not be sure whether the manipulation activated some other psychological experience (e.g., social hierarchy). This study only monitored emotional variables, and future studies are necessary to measure more other psychological variables that may be affected by movie excerpts. Furthermore, although the discriminant and predictive validity of the MMG has been convincingly demonstrated [42], evidence for its convergent validity is still missing. Future research should include other implicit (e.g., Picture Story Exercise [49]) or explicit measures of motivation (e.g., Personality Research Form [50]). This could further verify the efficacy of experimentally arousing motivations using movie excerpts. Second, previous researchers identified two types of power motives: personalized and socialized [4, 6, 51]. Personalized power is the desire for direct control or dominance of self-serving and antisocial goals. Individuals strongly motivated by personalized power tend to treat life as a “zero-sum game,” with a “me-against-the-world attitude” [4]. It is often impossible to care for the needs and feelings of others [51]. In this case, power and status are regarded as means of attaining profit. Socialized power, however, is characterized by helping others. This can be in instructing or supporting them, which is socially acceptable and constructive. It motivates people to attain status through prestige—gaining status through social influence and respect [52]. According to this distinction, personalized power motives are supposedly more susceptible to social context than socialized power motives. Performance driven by an individualized power motivation may transform from self-serving to altruistic behavior in a public situation. Future research should address this question. Third, in the dictator game, we used a stranger girl in need as the recipient of donations. However, the gender of the recipient is a considerable factor influencing helping behavior [53, 54]. Therefore, future research is needed to determine whether the recipient’s gender moderates prosocial donations. Finally, in this study, donation sizes in a modified dictator game were used as a measure of prosocial behavior. Future studies should expand this measurement to include other kinds of prosocial behavior.

## Conclusion

Whereas traditional approaches associate power motives with antisocial behavior and egotistical action, our findings suggest that arousing power motivations in certain contexts (i.e., social presence) can turn people away from selfishness and instead choose prosocial behaviors. The core requirement of the power motivation is to develop influence. To this end, people whose power motivation is activated may adopt different strategies according to different social situations. Public displays of selflessness and generosity are likely to bolster an individual’s reputation and status. This, in turn, makes it easier for them to attain power. From this perspective, socially preferable behavior of power motivated people in public may be less altruistic and more a strategy for gaining reputation and influence.

## Supporting information

S1 Data Raw data from the questionnaires and behavioral task. (CSV)
